# Scaffold Hopping and Structural Modification of NSC 663284: Discovery of Potent (Non)Halogenated Aminobenzoquinones

**DOI:** 10.3390/biomedicines12010050

**Published:** 2023-12-24

**Authors:** Nilüfer Bayrak, Belgin Sever, Halilibrahim Ciftci, Masami Otsuka, Mikako Fujita, Amaç Fatih TuYuN

**Affiliations:** 1 Department of Chemistry, Faculty of Science, Istanbul University, Fatih, İstanbul 34126, Turkey; nbayrak@istanbul.edu.tr; 2 Department of Pharmaceutical Chemistry, Faculty of Pharmacy, Anadolu University, Eskisehir 26470, Turkey; belginsever@anadolu.edu.tr; 3 Medicinal and Biological Chemistry Science Farm Joint Research Laboratory, Faculty of Life Sciences, Kumamoto University, Kumamoto 862-0973, Japan; hiciftci@kumamoto-u.ac.jp (H.C.); motsuka@gpo.kumamoto-u.ac.jp (M.O.); mfujita@kumamoto-u.ac.jp (M.F.); 4 Department of Drug Discovery, Science Farm Ltd., Kumamoto 862-0976, Japan; 5 Department of Molecular Biology and Genetics, Koc University, Istanbul 34450, Turkey

**Keywords:** scaffold hopping, anticancer activity, NSC 663284, quinones, leukemia, Abl TK, DNA, ADME, molecular docking

## Abstract

The development of new anticancer drugs is still ongoing as a solution to the unsatisfactory results obtained by chemotherapy patients. Our previous studies on natural product-based anticancer agents led us to synthesize a new series of Plastoquinone (PQ) analogs and study their anticancer effects. Four members of PQ analogs (**PQ1**–**4**) were designed based on the scaffold hopping strategy; the design was later completed with structural modification. The obtained PQ analogs were synthesized and biologically evaluated against different cancer genotypes according to NCI-60 screening in vitro. According to the NCI results, bromo and iodo-substituted PQ analogs (**PQ2** and **PQ3**) showed remarkable anticancer activities with a wide-spectrum profile. Among the two selected analogs (**PQ2** and **PQ3**), **PQ2** showed promising anticancer activity, in particular against leukemia cell lines, at both single- and five-dose NCI screenings. This compound was also detected by MTT assay to reveal significant selectivity between Jurkat cells and PBMC (healthy) compared to imatinib. Further in silico studies indicated that **PQ2** was able to occupy the ATP-binding cleft of Abl TK, one of the main targets of leukemia, through key interactions similar to dasatinib and imatinib. **PQ2** is also bound to the minor groove of the double helix of DNA. Based on computational pharmacokinetic studies, **PQ2** possessed a remarkable drug-like profile, making it a potential anti-leukemia drug candidate for future studies.

## 1. Introduction

The journey of lead structure(s) from preclinical studies to clinic trials in the traditional drug development strategy is tough, risky, and time-consuming; this process frequently lasts for decades and generally costs not less than US $1 billion. The discovery of lead structure(s) from lead optimization, which is by no means a simple task, is mostly the center of a medicinal and/or organic chemist’s effort [1]. Based on lead optimization, optimized drug candidates and improved pharmacophore models could be generated by numerous structure-activity relationship (SAR) investigations and physicochemical parameter optimizations. Large libraries that concentrate on arrays of bioisosteric replacements are designed and synthesized as part of this process in order to understand pertinent pharmacophore details. A bioisosteric replacement based on a physicochemical or topological basis is used to produce a new molecule with biological properties essentially comparable to those of the original substance. The toxicity, biological potency, and/or pharmacokinetic properties of the lead structure may be attenuated or strengthened by a bioisosteric replacement strategy [2].

Scaffold hopping approaches [3,4], natural product-inspired approaches [5,6], targeted protein degradation (PROTACs) [7,8], and computer-aided, particularly artificial intelligence (AI)-based design [9,10] are the most important examples of current drug design discovery approaches used in the world. The scaffold hopping approach, used as an effective alternative to develop a new lead structure, significantly speeds up drug development time in addition to lowering the risk of failure and reducing costs. Scaffold hopping was first invented by Schneider and colleagues in the late 20th century, and it takes its place in the drug development process [11]. The phrase “scaffold hopping” is widely used in the literature in drug development studies to reveal new lead candidates, from straightforward heterocyclic substitutions to topological structural modifications ranging from single atom replacements to complete structures. While the main core moiety is redesigned in the scaffold hopping strategy by replacing a larger moiety, the pendant group(s) in comparable positions are kept constant [12]. Many analogs generated by the scaffold hopping approach based on existing drugs, clinical candidates, bioactive agents [13,14], and natural products [15,16] have been approved by the US Food and Drug Administration (FDA) or are undergoing clinical trials [17,18]. Sampangine is a small molecule used extensively in investigations for its antimicrobial and antitumor potential. Inspired by the molecular backbone of Sampangine, Jiang et al. designed new analogs as antifungal lead compounds with facile synthesis, low toxicity, and strong inhibitory effects on fungal biofilms [19]. Chen et al. found that a simplified sampangine derivative has the ability to inhibit acetylcholinesterase (AChE), butyrylcholinesterase (BChE), and β-myeloid (Aβ) aggregation with increased water solubility and reduced toxicity [20].

**Figure 1:**
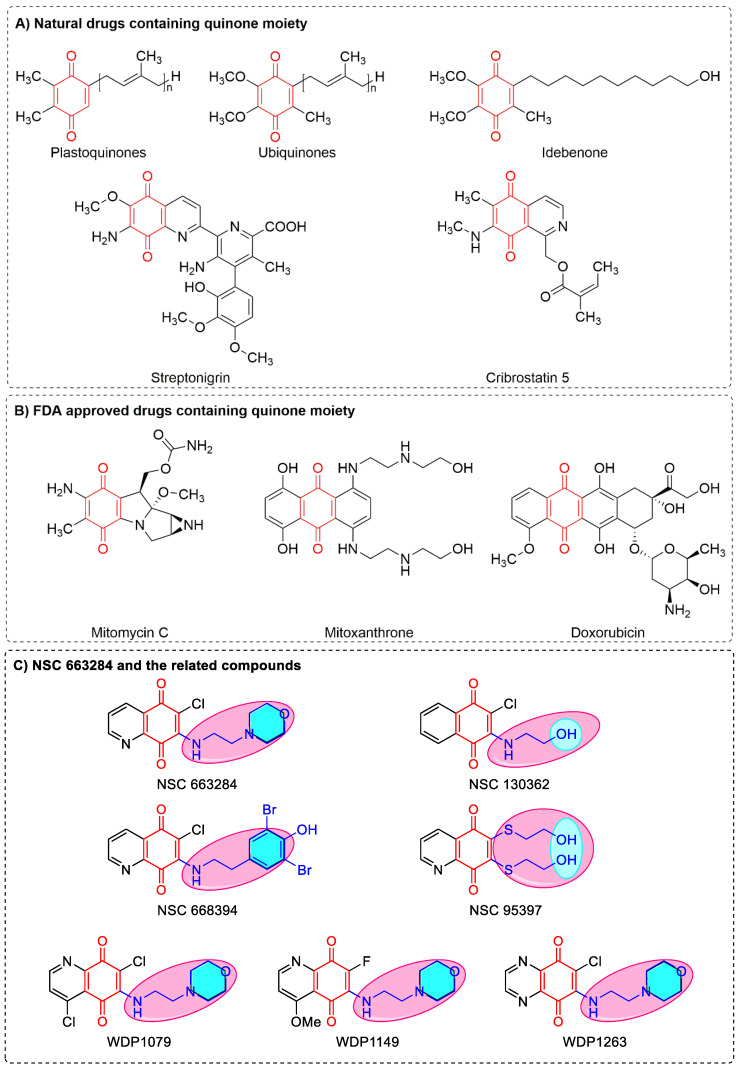
(**A**) Natural drugs containing quinone moiety; (**B**) FDA-approved drugs containing quinone moiety; and (**C**) NSC 663284 and the related compounds.

1,4-Quinone core has been identified as a privileged structural scaffold in various pharmaceuticals, natural products, and materials chemistry [21,22,23,24]. As a result, tremendous efforts have been put into the development of functionalized quinones [25,26]. Several important natural or synthetic molecules containing quinone core, especially 1,4-benzoquinone, are shown in Figure 1, such as Plastoquinones, Ubiquinones, Idebenone [27,28,29,30], Streptonigrin [31,32,33], Cribrostatin 5 [34,35], Mitomycin C [36,37], Mitoxantrone [38,39], and Doxorubicin [40]. On the other hand, quinolinequinone and naphthoquinone moieties are another two kinds of aromatic groups condensed to 1,4-quinone core structures with important biological properties [41,42,43,44]. Some representative important molecules with a quinone moiety are presented in Figure 1, namely NSC 663284 [45,46], NSC 668394 [47], NSC 130362 [44], and NSC 95397 [48], which were identified in the compound library of the National Cancer Institute (NCI). Both of them (NSC 663284 and NSC 668394) showed promising anticancer potency, in particular effective cell division cycle 25 protein inhibitors with low IC_50_ values. Based on the structure of the lead molecule (NSC 663284), Keinan et al. designed, synthesized, and evaluated new analogs (WDP1079, WDP1149, and WDP1263) of NSC 663284 with the Linear Combination of Atomic Potentials (LCAP) methodology. When analyzing their design, one can identify some modifications of NSC 663284, such as the substituent effect (chlorine atom), the additional nitrogen atom on quinolinequinone, and the position of the amino moiety attached to the quinolinequinone within WDP1079 and WDP1263. On the other hand, we can also define the substituent effect (methoxy group)/fluorine atom on quinolinequinone and the position of the amino moiety attached to the quinolinequinone within WDP1149. This is not surprising; the structure of the amino moiety (morpholinoethyl amino) and the quinone moiety (quinolinequinone) are the common structural features within all three potent analogs [49]. In recent years, Tang et al. conducted a comprehensive structural modification on the quinolinequinone moiety in addition to the linker and polar tail of the amino moiety based on the reference molecule [50]. Apart from this, Narwanti et al. mainly focused on the quinone moiety by keeping the amino moiety (morpholinoethyl amino). Amino-substituted quinolinequinones, isoquinolinequinones, and naphthoquinones with a nitro group or a halogen atom (bromo, chloro, or iodo) were designed and discovered as potent CDC25 inhibitors [51].

Given the important roles that NSC 663284 plays in medicinal chemistry, in particular in cancer therapy, with the intention of expanding the scarce collection of NSC 663284 analogs, we engaged in this molecule and its moieties as a starting point for our research. Thus, in order to develop more selective and potent therapeutic agents, we designed and synthesized some (non)halogenated Plastoquinone (PQ) analogs as NSC 663284 analogs and revealed the anticancer profile of the obtained molecules in addition to the docking studies.

## 2. Materials and Methods

### 2.1. Chemistry

All reagents, solvents, and corresponding substituted amines were commercially obtained from suppliers and were used as received without any further purification. Thin layer chromatography (TLC) analysis of crude reaction mixtures or pure samples was performed using aluminum-based Merck KGaA (silica gel 60 F254) TLC plates and visualized under UV light (254 nm). Column chromatography was carried out on a glass column by using the wet-packed method with silica gel 60 (Merck (Darmstadt, Germany), 63–200 µm particle-sized, 60–230 mesh). ^1^H and ^13^C NMR spectra were recorded at 500 MHz and 125 MHz on a Varian^UNITY^ INOVA spectrometer in CDCl_3_ as solvent, respectively. Chemical shifts (δ) are reported in ppm. Coupling constants (J) are reported in Hz. IR spectra were obtained from molecules in neat form with a Quest ATR (Attenuated Total Reflectance) accessory with a diamond crystal puck using a Shimadzu IRTracer-100 Fourier Transform Infrared Spectrophotometer (FTIR). High-resolution mass spectra (HRMS) were obtained with a Waters SYNAPT G1 MS using an electrospray ionization technique.

### 2.2. Synthesis of Halogenated 1,4-Benzoquinones (**4**–**6**)

The precursors 2,3-dibromo-5,6-dimethyl-1,4-benzoquinone (**4**) [52], 2,3-diiodo-5,6-dimethyl-1,4-benzoquinone (**5**) [53], and 2,3-dichloro-5,6-dimethyl-1,4-benzoquinone (**6**) [54] were synthesized using the reported method in the literature.

#### 2.2.1. Brominated 1,4-Benzoquinones (**4**)

Approximately 2,3-Dimethylhydroquinone (**1**, 1.0 g) dissolved in 5 mL of glacial acetic acid was mixed with 0.35 mL of Br_2_ in 5 mL of glacial acetic acid. After stirring for 30 min, the red-colored solution was poured into a beaker containing 50 mL of water. A white solid (**2**, 0.33 g, 33% yield) was separated by vacuum filtration and dried at room temperature. After that, 0.33 g of **2** in 3 mL of glacial acetic acid was mixed with 0.1 mL of Br_2_ in 3 mL of glacial acetic acid. Upon pouring into the 50 mL of water after 30 min, a violet solid (**3**, 0.44 g) was obtained by filtration. At the oxidation step, 0.44 g of **3** was dispersed in 5 mL of sodium hypochlorite (1.5% bleach) and stirred for 1 day. Chromatographic separation gave 2,3-dibromo-5,6-dimethyl-1,4-benzoquinone (**4**) [52] as a brown solid.

#### 2.2.2. Iodinated 1,4-Benzoquinones (**5**)

Approximately 2,3-Dibromo-5,6-dimethyl-1,4-benzoquinone (**4**, 0.5 g) and sodium iodide (0.7 g) were strictly refluxed for 1 day in freshly distilled ethyl methyl ketone. After evaporating the solvent in vacuo, the residue was suspended in acetone and poured into water. The 2,3-diiodo-5,6-dimethyl-1,4-benzoquinone (**5**) [53] as red-brown crystals were filtered off, washed with water, and recrystallized from alcohol with a 45% yield.

#### 2.2.3. Chlorinated 1,4-Benzoquinones (**6**)

The mixture was obtained by adding 5 mL of concd. HNO_3_ in small portions to a stirred suspension of 2,3-dimethylhydroquinone (**1**) in 15 mL of concd. HCl at 80–90 °C was stirred at room temperature for 2 h and was extracted with 25 mL of diethyl ether. The collected extracts were evaporated and crystallized in EtOH, which afforded 2,3-dichloro-5,6-dimethyl-1,4-benzoquinone (**6**) [54] as an orange powder.

### 2.3. Synthesis of PQ Analogs

#### 2.3.1. Procedure for the Preparation of PQ Analog (**PQ1**)

To a solution of the 4-(2-aminoethyl)morpholine (2 equiv.) and 2,3-dimethylhydroquinone (**1**, 1 equiv.) in methanol (12 mL), sodium iodate (3 equiv.) in water (12 mL) was added at room temperature and stirred for 12–24 h. The progress of the reaction was monitored by TLC. After the completion of the reaction, the solution was extracted, diluted with CHCl_3_ (30 mL) and distilled water (30 mL), and the combined organic layers were washed with water (3 × 30 mL), dried over anhydrous CaCl_2_, and concentrated under vacuum. Finally, the residue was purified by using column chromatography over silica gel to provide the **PQ1**.

##### 2,3-Dimethyl-5-((2-morpholinoethyl)amino)-1,4-benzoquinone (**PQ1**)

Obtained target analog (**PQ1**) as a light brown. Yield: 27%. FTIR (ATR) υ (cm^−1^): 3288 (NH), 2970, 2855, 2812 (CH_aliphatic_), 1640, 1570 (>C=O), 1500, 1341, 1300, 1273, 1146, 1115, 1071. ^1^H NMR (500 MHz, *CDCl_3_*) *δ* (ppm): δ 7.03 (br s, 1H, NH), 5.22 (s, 1H, -CH_quinone_), 3.76–3.71 (m, 4H, -OCH_2_-), 3.20 (m, 2H, -NH-CH_2_-), 2.66 (t, *J* = 6.0 Hz, 2H, -CH_2_-N<), 2.49 (s, 4H, -NCH_2_-), 2.09 (s, 6H, -CH_3_). ^13^C NMR (125 MHz, *CDCl_3_*) *δ* (ppm): 179.56, 179.17 (>C=O), 150.36, 147.97, 110.38, 92.37 (C_quinone_), 66.80 (OCH_2_), 55.63 (NCH_2_), 53.20, 38.47 (-CH_2_-), and 10.16 (CH_3_). HRMS(+ESI) *m/z* calcd for C_14_H_21_N_2_O_3_ [M + H]^+^: 265.1552; found: 265.1606.

#### 2.3.2. General Procedures for the Preparation of PQ Analogs (**PQ2**–**4**)

To a solution of the corresponding halogenated dimethyl-1,4-benzoquinones (**4**, **5**, or **6**) (1.0 equiv) in methanol (10 mL), 4-(2-aminoethyl)morpholine (1.2 equiv) was added to a stirred solution in methanol (5 mL) at room temperature and stirred for 8–16 h until consumption of the precursor. The progress of the reaction was monitored by TLC. After the completion of the reaction, the solution was diluted with CHCl_3_ (30 mL) and distilled water (30 mL), and the combined organic layers were sequentially washed with water (3 × 30 mL) again, dried over anhydrous CaCl_2_, and concentrated under vacuum. Finally, the residue was purified by using column chromatography over silica gel to provide the corresponding **PQ**.

##### 2-Bromo-5,6-dimethyl-3-((2-morpholinoethyl)amino)-1,4-benzoquinone (**PQ2**)

Obtained target analog (**PQ2**) as a reddish sticky oil. Yield: 35%. FTIR (ATR) υ (cm^−1^): 3248 (NH), 2955, 2901, 2859, 2835 (CH_aliphatic_), 1649, 1626, 1589 (>C=O), 1495, 1456, 1373, 1298, 1267, 1155, 1076, 1038, 1024, 957. ^1^H NMR (500 MHz, *CDCl_3_*) *δ* (ppm): δ 6.67 (br s, 1H, NH), 3.87 (dd, *J* = 11.3, 5.6 Hz, 2H, -NH-CH_2_-), 3.81–3.71 (m, 4H, -OCH_2_-), 2.64 (t, *J* = 5.8 Hz, 2H, -CH_2_-N<), 2.52 (s, 4H, -NCH_2_-), 2.09 (d, *J* = 1.2 Hz, 3H, -CH_3_), 1.99 (d, *J* = 1.2 Hz, 3H, -CH_3_). ^13^C NMR (125 MHz, *CDCl_3_*) *δ* (ppm): 182.36, 178.47 (>C=O), 144.88, 143.72, 135.92 (C_quinone_), 66.86 (OCH_2_), 56.76 (NCH_2_), 52.95, 40.70 (-CH_2_-), 13.84, 12.18 (CH_3_). HRMS(+ESI) *m/z* calcd for C_14_H_20_N_2_O_3_Br [M + H]^+^: 343.0657; found: 343.0641.

##### 2-Iodo-5,6-dimethyl-3-((2-morpholinoethyl)amino)-1,4-benzoquinone (**PQ3**)

Obtained target analog (**PQ3**) as a reddish sticky oil. Yield: 62%. FTIR (ATR) υ (cm^−1^): 3198 (NH), 2953, 2812 (CH_aliphatic_), 1655, 1560 (>C=O), 1445, 1368, 1296, 1263, 1242, 1217, 1077, 1057, 1034, 1018. ^1^H NMR (500 MHz, *CDCl_3_*) *δ* (ppm): δ 6.72 (br s, 1H, NH), 3.86 (d, *J* = 5.5 Hz, 2H, -NH-CH_2_-), 3.81–3.69 (m, 4H, -OCH_2_-), 2.63 (t, *J* = 5.7 Hz, 2H, -CH_2_-N<), 2.52 (s, 4H, -NCH_2_-), 2.12 (d, *J* = 1.1 Hz, 3H, -CH_3_), 2.00 (t, 3H, -CH_3_). ^13^C NMR (125 MHz, *CDCl_3_*) *δ* (ppm): 181.66, 179.28 (>C=O), 149.43, 142.73, 135.87, 109.99 (C_quinone_), 66.98 (OCH_2_), 56.47 (NCH_2_), 52.86, 41.44 (-CH_2_-), 14.40, 12.13 (CH_3_). HRMS(+ESI) *m/z* calcd for C_14_H_20_N_2_O_3_I [M + H]^+^: 391.0519; found: 391.0517.

##### 2-Chloro-5,6-dimethyl-3-((2-morpholinoethyl)amino)-1,4-benzoquinone (**PQ4**)

Obtained target analog (**PQ4**) as a reddish sticky oil. Yield: 52%. FTIR (ATR) υ (cm^−1^): 3281 (NH), 2936, 2853 (CH_aliphatic_), 1655, 1585 (>C=O), 1489, 1458, 1371, 1294, 1261, 1227, 1167, 1144, 1088, 1024. ^1^H NMR (500 MHz, *CDCl_3_*) *δ* (ppm): δ 6.55 (br s, 1H, NH), 3.84 (dd, *J* = 11.5, 5.8 Hz, 2H, -NH-CH_2_-), 3.78–3.68 (m, 4H, -OCH_2_-), 2.62 (t, *J* = 6.0 Hz, 2H, -CH_2_-N<), 2.50 (s, 4H, -NCH_2_-), 2.15–2.01 (m, 3H, -CH_3_), 1.98 (d, *J* = 1.2 Hz, 3H, -CH_3_). ^13^C NMR (125 MHz, *CDCl_3_*) *δ* (ppm): 182.59, 178.92 (>C=O), 143.90, 142.45, 135.99, 109.99 (C_quinone_), 66.88 (OCH_2_), 56.93 (NCH_2_), 53.01, 40.37 (-CH_2_-), 13.46, 12.15 (CH_3_). HRMS(+ESI) *m*/*z* calcd for C_14_H_20_N_2_O_3_Cl [M + H]^+^: 299.1162; found: 299.1153.

### 2.4. Biological Evaluation

#### 2.4.1. In Vitro Single-Dose Anticancer Screening by NCI

The obtained PQ analogs (**PQ1**–**4**) were submitted to the National Cancer Institute (NCI), Bethesda, USA, and as per the standard protocol of the NCI, all compounds were evaluated for their antiproliferative activity at a single-dose assay (10 µM concentration in DMSO) on a panel of 60 cancer cell lines derived from leukemia, melanoma, non-small cell lung, colon, CNS, ovarian, renal, prostate, and breast cancers as per protocol. Tested compounds were added to the microtiter culture plates, followed by incubation for 48 h at 37 °C. Sulforhodamine B (SRB), a protein-binding dye, was used for end point determination. The percent growth of the treated cells was determined in comparison to the untreated control cells, and the results of each tested compound were reported. Data from one-dose experiments pertains to the percentage growth at 10 μM [55,56,57].

#### 2.4.2. In Vitro Five-Dose Anticancer Screening by NCI

A serial 5 × 10-fold dilution from an initial DMSO stock solution was performed prior to incubation at each individual concentration. The selected PQ analogs (**PQ2** and **PQ3**) were then elevated by DTP-NCI for a higher testing level to determine three dose-response parameters (GI_50_, TGI, and LC_50_) for each cell line after establishing a dose-response curve from five different concentrations (0.01, 0.1, 1, 10, and 100 µM) for **PQ2** and **PQ3**.

#### 2.4.3. Cell Culture, Drug Treatment, and MTT Assay

In the current study, RPMI 1640 (Wako Pure Chemical Industries, Osaka, Japan) medium was used for cultures of the Jurkat cell line and PBMCs (Precision Bioservices, Frederic, MD, USA). The medium contained 10% fetal bovine serum (FBS) (Sigma Aldrich, MO, USA) and 89 μg/mL streptomycin (Meiji Seika Pharma, Tokyo, Japan). These cells were incubated at 37 °C in a humidified 5% CO_2_ atmosphere and plated onto 24-well and 96-well tissue plates (Iwaki brand Asahi Glass Co., Chiba, Japan) with 2 × 10^4^ (for Jurkat cells) and 1 × 10^6^ (for PBMC) cells/mL density, and then incubated for 24 h (the optimum cell number was quantified as associated with our previous study) [58,59]. The stock solution of **PQ2** and imatinib in concentrations ranging from 0.1 to 10 mM was prepared in DMSO (Wako Pure Chemical Industries) and further diluted with fresh culture medium. In order to prevent any effect on cell viability, the final DMSO concentration was adjusted to 1% [58,59].

Based on the previously explained procedures in the literature [58,59] to determine the cytotoxic effects of **PQ2** on Jurkat cell lines and PBMCs compared to imatinib, the MTT (Dojindo Molecular Technologies, Kumamoto, Japan) assay was assessed. These cells were treated with MTT solution, and then they were incubated for an additional 4 h after 24 h of exposure to different concentrations (1–100 μM) of **PQ2** and imatinib at 37 °C. Following the additional incubation, the supernatants were discarded, and the formazan crystals formed by MTT metabolism were solubilized by the addition of 100 μL DMSO to each well. The absorbance for each well was measured on a plate reader, the Infinitive M1000 (Tecan, Mannedorf, Switzerland), at a wavelength of 550 nm with background subtraction at 630 nm. All experiments were carried out in triplicate, and the IC_50_ values, the drug concentrations reducing absorbance to 50% of control values, were determined to be related to the results of the MTT assay [58,59].

### 2.5. Computational Studies

#### 2.5.1. Molecular Docking Studies

The crystal structures of Abl TK and DNA were obtained from the RSCB database (PDB IDs: 2GQG [60] for Abl TK and 2GWA [61] for DNA). The raw file for the docking analysis was prepared by the PrepWizard module of Maestro. The missing chains were added automatically by Prime, and the protonation state was calculated by PropKa at physiological pH. The receptor–ligand complex was minimized by the Optimized Potential Liquid Simulations (OPLS_2005) force field. **PQ2**, dasatinib, and imatinib were drawn and cleaned in the Maestro workspace and prepared with energy minimization using the OPLS_2005 force field at physiological pH using the LigPrep module. Grid generation and Glide/SP docking protocols were carried out for **PQ2**, dasatinib, and imatinib [58,59,62,63,64].

#### 2.5.2. In-Silico ADME Estimation

The key pharmacokinetic properties of **PQ2** were predicted by the QikProp module of the Maestro QikProp [65] and SwissADME [66] tools.

## 3. Results

### 3.1. Design and Synthesis

**Figure 2:**
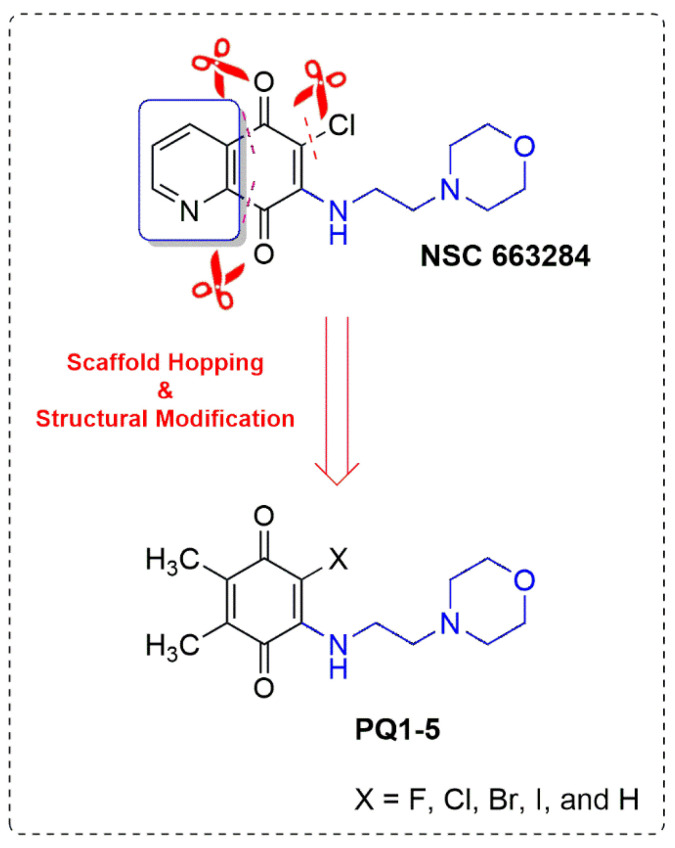
Design strategy for the PQ analogs.

To determine the new lead structure as NSC 663284 analogs that are mainly selective anticancer efficacy and safety in normal cells, our design strategy is based on our previous findings that our group reported successful examples of halogenated and nonhalogenated PQ analogs, namely 2,3-dimethyl-1,4-benzoquinones [67,68,69,70,71]. Based on the aforementioned studies of our group and literature, we envisioned that the pyridinyl moiety of NSC 663284 could be replaced by dimethyl groups (by ring-opening and simplification to generate a new core structure) using a scaffold hopping approach to design novel PQ analogs that could be a potential inhibitor in cancer therapy, as shown in Figure 2. Our design strategy continued with structural modification by replacing the chlorine atom with other halogen and hydrogen atoms.

**Scheme 1:**
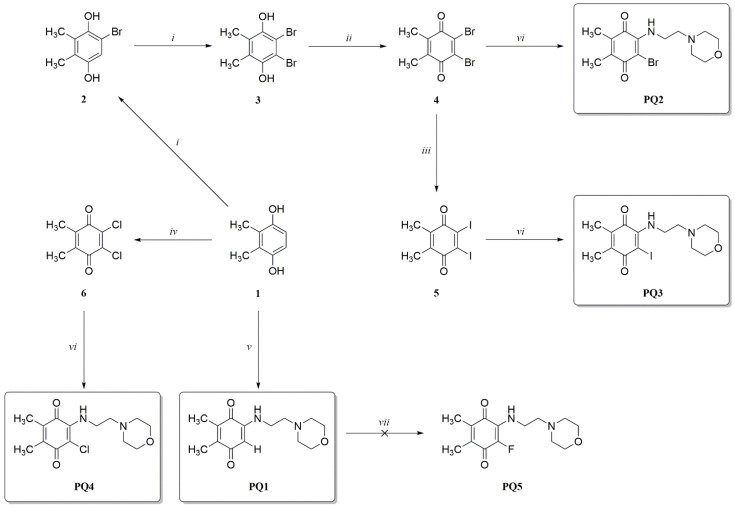
Reagents and conditions: (*i*) Br_2_, CH_3_COOH, rt, 30 min; (*ii*) sodium hypochlorite (1.5% bleach); (*iii*) NaI, MEK, reflux, overnight; (*iv*) HNO_3_/HCl, 10 min, 90 °C; (*v*) 4-(2-aminoethyl)morpholine, NaIO_3_, H_2_O/MeOH, rt, 24 h; (*vi*) 4-(2-aminoethyl)morpholine, MeOH, rt, 8–16 h; (*vii*) Selectfluor, CH_3_CN, rt.

The chemical synthesis of nonhalogenated (**PQ1**) and halogenated PQ analogs (**PQ2**–**5**) is depicted in Scheme 1, starting from commercially available dimethylhydroquinone (**1**). Intermediate (**4**) required for the preparation of the brominated PQ analog (**PQ2**) and iodinated PQ analog (**PQ3**) was synthesized from dimethylhydroquinone (**1**) *via* dibromination in two steps, followed by the oxidation with sodium hypochlorite according to reported literature [52]. Iodination of the precursor (**4**) with NaI in ethyl methyl ketone gave another important precursor (**5**) for the preparation of the iodinated PQ analog (**PQ3**) [53]. Dichlorobenzoquinone (**6**) was prepared from the chlorooxidation of dimethylhydroquinone (**1**) by using HNO_3_/HCl at 90 °C, according to the literature [54]. The nonhalogenated PQ analog (**PQ1**) was synthesized by a one-pot reaction between dimethylhydroquinone (**1**) and 4-(2-aminoethyl)morpholine in the presence of NaIO_3_ at room temperature, according to the literature [72]. The halogenated PQ analogs (**PQ2**–**4**) were obtained by reacting the intermediates (**4**, **5**, and **6**, respectively) with 4-(2-aminoethyl)morpholine by substitution reaction in the MeOH. Unfortunately, the synthesis of another target PQ analog (**PQ5**) from **PQ1** with selectfluor in acetonitrile could not be obtained.

The PQ analogs (**PQ1**–**4**) have shown similar characteristic bands and peaks in the different spectroscopic analyses. All high-resolution mass spectrometric experiments (ESI-HRMS) confirmed the molecular mass and purity. According to infrared spectra, amino moieties exhibited absorption bands from vibrations in the region of 3200 cm^−1^ corresponding to the NH group. The absorption bands of the carbonyl group of the quinone in the region 1650 cm^−1^ were observed. The signal of the amino proton signal was detected at around 6.70 ppm. It is established from the data of the ^1^H NMR spectrum of the nonhalogenated PQ analog (**PQ1**) that the hydrogen corresponding to the quinone moiety resonated as a singlet at 5.22 ppm. Hydrogens corresponding to the methyl groups in the quinone moiety were observed in the region at around 2.00 ppm. The two methylene protons attached to the morpholine moiety were observed at around 3.77 and 2.60 ppm. In the ^13^C NMR spectra, it was observed that the two signals related to carbonyl carbons of the quinone moiety were at around 180 ppm, as well as the ethylene groups at around 50 and 40 ppm. Additionally, signals at around 13, 55, and 65 ppm are present, which is typical of methyl carbons attached to the quinone moiety and methylene carbons within the morpholine, respectively. The spectra of the PQ analogs were given in Supplementary File as Figures S1–S12.

### 3.2. Cell-Based Anticancer Screening for PQ Analogs

#### 3.2.1. Single-Dose Screening on NCI 60 Cancer Cells

The obtained target analogs (**PQ1**–**4**) were submitted and selected by the National Cancer Institute (NCI, Bethesda, MD, USA (http://www.dtp.nci.nih.gov, accessed on 11 May 2022)) for evaluation at a single concentration (10 µM) under the drug discovery program of the NCI towards a panel of sixty cancer cell lines of nine human cancer cell types (leukemia, melanoma, non-small-cell lung, colon, central nervous system, ovarian, renal, prostate, and breast cancer) according to NCI protocol. The preliminary screening results are growth percentages relative to the no-drug control and to the number of cells at the zero-time point. Table 1 summarizes the findings as the growth percentage (GP) and lethality (values less than 0) of the treated cells. The percentage growth inhibition (GI%) values were obtained using the mean growth percentages (100—Growth percentage). Two PQ analogs, **PQ1** and **PQ4**, exhibited generally weak to no growth inhibition against most of the cancer cell lines. **PQ4** caused good growth inhibition activity in a few cell lines, i.e., CCRF-CEM, HCT-116, and LOX IMVI (73.84, 62.03, and 76.76%, respectively) in some instances. The profiles of the remaining two PQ analogs (**PQ2** and **PQ3**) were entirely different. Some cell lines were extremely sensitive to the aforementioned PQ analog, with up to 99% growth inhibition values. Regarding the leukemia subpanel, these two analogs displayed anticancer profiles ranging from modest to potent activity (growth inhibition ≥ 41.41% and up to 99.72%) on five types of leukemia cell lines. Both compounds (**PQ2** and **PQ3**) revealed excellent potential inhibition activity on the K-562 cell line, with 99.01 and 94.76%, respectively. **PQ2** exhibited significant inhibitory effects on the MOLT-4 cell line from the leukemia panel with 99.72% inhibition, while **PQ3** exerted prominent potency against HL-60(TB) of the leukemia cell line with 89.74% inhibition. Regarding the non-small cell lung cancer subpanel, results indicated that **PQ2** has modest anticancer activity on the EKVX and HOP-92 cell lines. Notably, **PQ3** showed growth inhibition potency against NCI-H23 and NCI-H226 cells, with 91.86% and 72.12% inhibition, respectively. **PQ3** inhibited the growth of HCT-116 and HT29 colon cancer cell lines strongly, with 85.45% and 84.50% growth inhibition, respectively. In addition, the growth of the SW-620 cell line belonging to the colon cancer subpanel and the growth of the SF-539 and U251 cell lines belonging to the CNS cancer subpanel were inhibited by **PQ2** with 67.29%, 67.94%, and 55.22% growth inhibition, respectively. Remarkably, **PQ2** displayed maximum sensitivity towards OVCAR-5 and OVCAR-8 cell lines from the ovarian cancer subpanel, with 96.00% and 83.69% inhibition in tumor cell growth, respectively. In addition, **PQ3** also exerted an anticancer profile against OVCAR-3, OVCAR-4, and OVCAR-5 cell lines from the same subpanel (98.88%, 65.45%, and 94.51%, respectively). Both **PQ2** and **PQ3** were found to be inactive and/or weakly active against renal and prostate cancer cell lines. In addition to this, **PQ3** was also found inactive and/or weakly active against some of the other cancer cell lines, such as melanoma and breast cancer cell lines. **PQ2** displayed growth inhibition moderately against M14 and UACC-62 cell lines, which belong to the melanoma subpanel, with 62.67% and 60.63% growth inhibition, respectively. **PQ2** was also active against the MCF-7 breast cancer cell line with 89.67% growth inhibition. Upon focusing on the screening findings, the best growth inhibition profile spectrum was shown by bromo and iodo-substituted PQ analogs (**PQ2** and **PQ3**, respectively). 

#### 3.2.2. Five-Dose Screening on NCI 60 Cancer Cell Lines Assay

Two PQ analogs (**PQ2** and **PQ3**) as lead analogs were advanced to the five-dose screening stage by the NCI for further exploration against a panel of 60 human cancer cell lines. Each analog was evaluated against each cell line at five 10-fold serial dilutions (100, 10, 1.0, 0.1, and 0.01 μM) after 48 h of treatment after satisfying the pre-determined threshold inhibition criteria (GI_50_ (the concentration at which 50% of growth inhibitory action), TGI (total growth inhibition), and LC_50_ (the concentration at which 50% of cancer cell death)) of NCI in the NCI single-dose assay shown in Table 2 (all in µM) [55,56].

**Figure 3:**
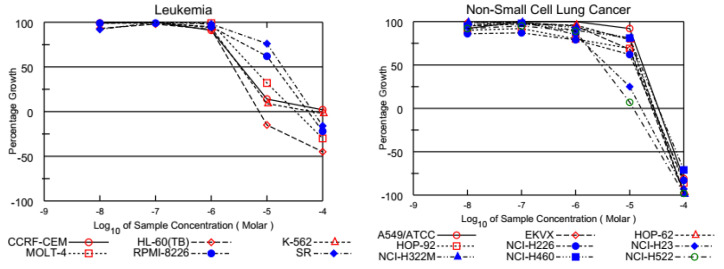

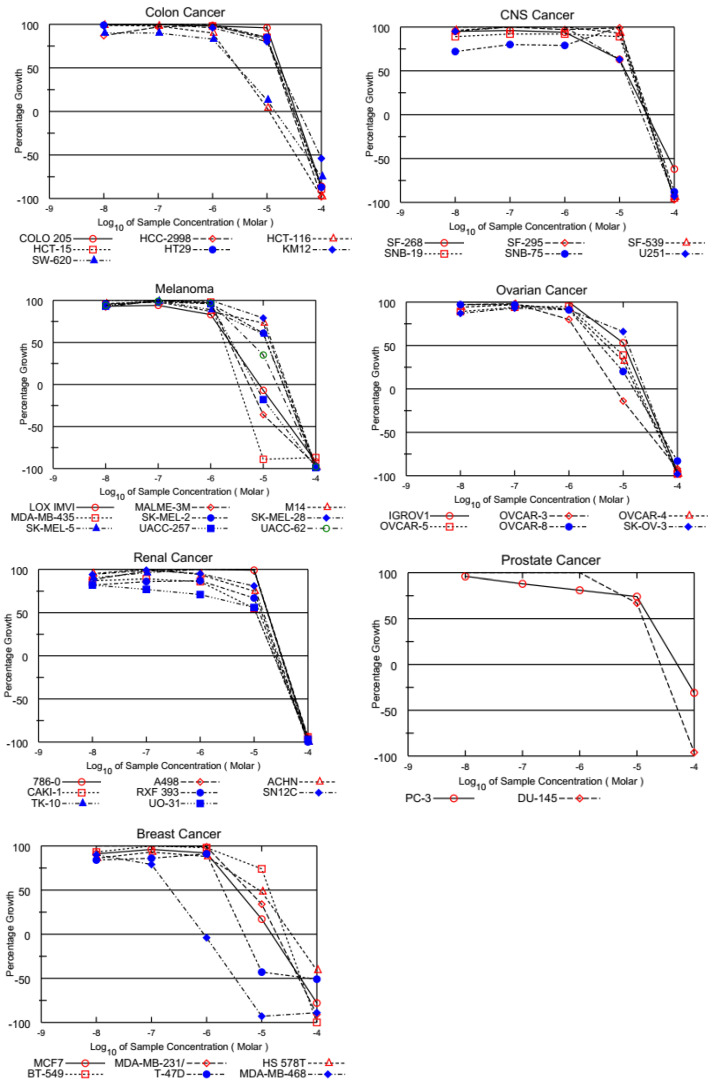
Graphical presentation of growth inhibition of **PQ2** at five-dose concentrations.

Table 2 shows that the GI_50_ values for the tested PQ analogs were less than 20 μM against most cell lines. The analysis of the GI_50_ values revealed that leukemia, melanoma, ovarian cancer, and breast cancer cell lines were particularly responsive to treatment with **PQ2** and **PQ3** with GI_50_ values of 0.22–9.04 μM by maintaining micromolar activity against all the tested cell lines. The best anticancer potency in specific subpanels was observed for the melanoma MDA-MB-435 cell line (GI_50_ value 1.81 μM) and the breast cancer T-47D (GI_50_ value 2.01 μM) and MDA-MB-468 cell lines (GI_50_ value 0.22 μM) for **PQ2**. This analog also demonstrated superior cytotoxic activity against T-47D and MDA-MB-468 cell lines, with TGI values of 4.74 µM and 0.885 µM, respectively. In addition, **PQ3** showed remarkable anticancer activity against the melanoma cell lines (MALME-3M and MDA-MB-435 with GI_50_ values of 1.71 µM and 1.72 μM, respectively) and the breast cancer cell lines (MCF7 and MDA-MB-468 with GI_50_ values of 1.80 and 1.21 μM, respectively). Furthermore, both PQ analogs displayed valuable inhibition against most of the tested cancer cell lines, with a GI_50_ below 10.00 µM. Comparable potency was also exerted against leukemia (GI_50_ 2.46–5.35 μM) and breast cancer (GI_50_ 0.22–9.04 μM) subpanels, with the exception (GI_50_ > 10 μM) of the RPMI-8226 and SR from the leukemia subpanel and BT-549 from the breast cancer subpanel cell lines, respectively. Both PQ analogs also had overall potency with high selectivity against the melanoma and ovarian cancer subpanels (GI_50_ values of 1.81–5.78 μM and 1.71–8.19 μM, respectively). Both analogs also possessed notable TGI values against the mentioned ovarian cancer cell lines in the range of 6.51–19.30 µM. CNS, renal, and prostate cancer cell lines were not sensitive to PQ analogs, with an average GI_50_ of over 10.00 μM, excluding **PQ3**, with a GI_50_ value of 2.31 uM against the DU-145 cell line of the prostate cancer subpanel. Some of the GI_50_ of two analogs against the melanoma cell lines, LOX IMVI, MALME-3M, MDA-MB-435, SK-MEL-28, UACC-257, and UACC-62, were under 10.00 μM. TGI values less than 20.0 μM were obtained for some of the cell lines. Moreover, good TGI values ranging from 3.18 to 18.40 µM were obtained against the mentioned melanoma cell lines. Herein, **PQ2** could be selected regarding the protocol of the NCI for the evaluation of its anticancer potency against a broad range of cancer cell types, in particular leukemia cancer cell lines, at five doses with significant GI_50_, TGI, and LC_50_ values. This finding pointed out that bromo substituted within the quinone moiety contributed to the anticancer effects of **PQ2**. Figure 3 depicts all five-dose response curves for **PQ2** against a panel of sixty human cancer cell lines.

NCI screening does not involve cytotoxicity studies against healthy cells to determine the selectivity of anticancer activity. Therefore, we further examined the cytotoxic effects of **PQ2** between the Jurkat human leukemic T-cell line and human peripheral blood mononuclear cells (PBMC) (healthy) *via* the MTT (3-(4,5-dimethyl-2-thiazolyl)-2,5-diphenyltetrazolium bromide) assay in comparison with imatinib. The IC_50_ values of **PQ2** for the Jurkat cell line and PBMC were found to be 2.94 ± 1.72 μM and 12.62 ± 4.07 μM, respectively, compared to imatinib (IC_50_ = 7.11 ± 1.18 μM for Jurkat cells and 25.14 ± 6.41 μM for PBMC). The more significant Selectivity Index (SI) of **PQ2** (SI = 4.29) than imatinib (SI = 3.54), which was calculated between the IC_50_ values of PBMC and Jurkat cells, indicated its selective anti-leukemic effects (Table 3).

**PQ2** was identified as the most effective and selective anticancer agent based on in vitro experiments, and this derivative showed the most prominent activity against leukemia cells at both single- and five-dose screenings. Therefore, we performed further in silico studies focusing on leukemia.

The human Philadelphia (Ph) chromosome, the result of the translocation between chromosomes 9 and 22, constitutes a Bcr-Abl (breakpoint cluster region–Abelson leukemia) hybrid gene. This gene expression results in Bcr-Abl tyrosine kinase (TK) activity, the hallmark of chronic myeloid leukemia (CML). The discovery of the main role of Abl TK in CML has pioneered the design and development of well-known Abl TK inhibitors, including imatinib and dasatinib. The mechanism of action of these inhibitors is binding to the ATP-binding cleft of Abl TK, restraining Abl TK autophosphorylation, and subsequent stimulation of downstream cascades that may result in proliferation, suppression of apoptosis, metastasis, and angiogenesis [73,74,75].

**Figure 4:**
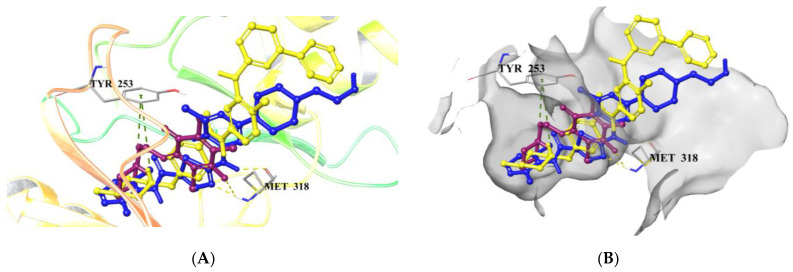
Docking poses of **PQ2** along with dasatinib and imatinib in the ATP binding cleft of Abl TK (PDB code: 2GQG) (**A**) Ribbon presentation (**B**) Surface presentation (Yellow dashes: hydrogen bonding, green dashes: π-cation) (**PQ2**, dasatinib, and imatinib were colored in maroon, blue, and yellow, respectively).

**Figure 5:**
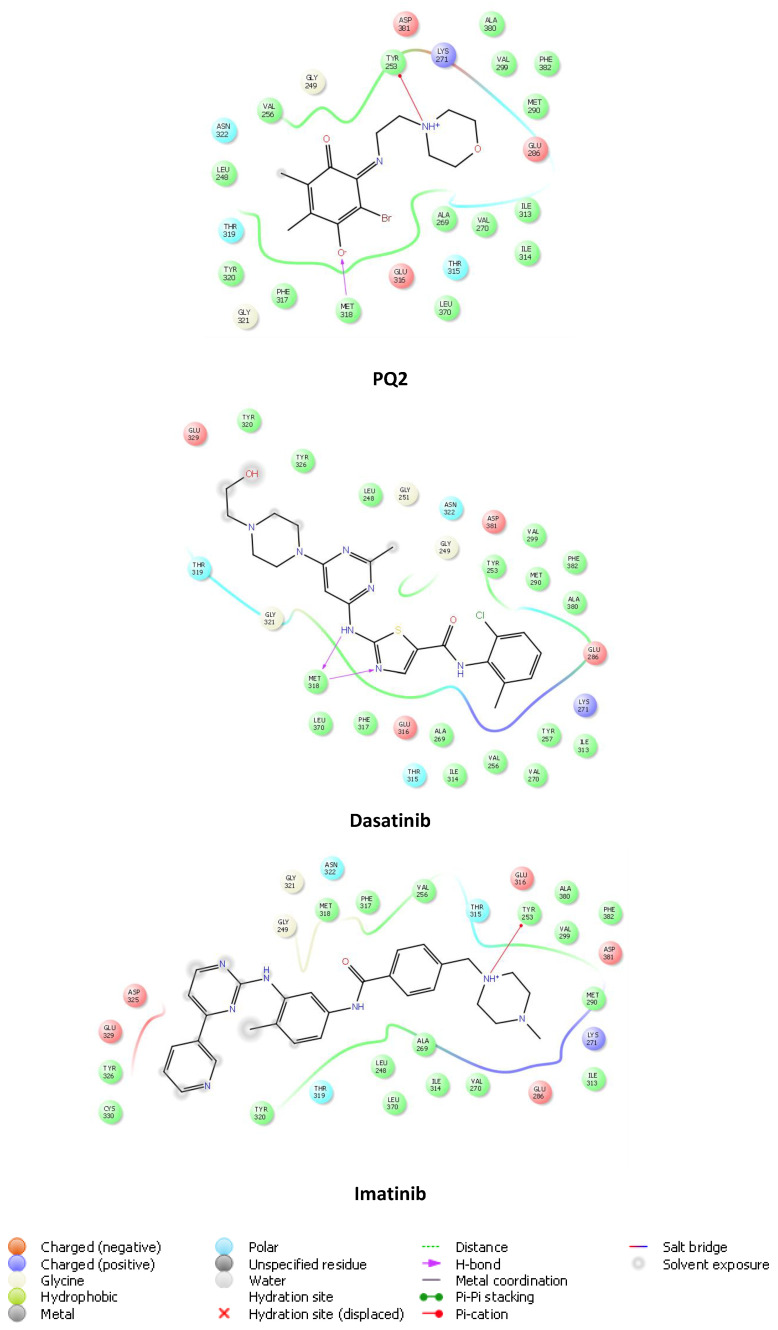
Docking interactions of **PQ2** along with dasatinib and imatinib in the ATP binding cleft of Abl TK (PDB code: 2GQG).

We performed molecular docking studies in order to understand the interactions of compound **PQ2** in the ATP-binding cleft of the Abl TK. The molecular docking studies were assessed in comparison with imatinib and dasatinib (PDB ID: 2GQG) [60]. Results showed that **PQ2** was able to occupy the binding region with a high affinity along with dasatinib and imatinib (Figure 4A,B). **PQ2** formed a hydrogen bond with Met318 and a π-cation with Tyr253. Dasatinib established two key hydrogen bonds with Met318, whereas imatinib formed a π-cation with Tyr253. This outcome indicated that **PQ2** possessed a similar binding profile to dasatinib and imatinib (Figure 5). The docking score of compound **PQ2** was found to be −7.783 kcal/mol, compared to dasatinib (−9.013 kcal/mol) and imatinib (−8.218 kcal/mol).

**Figure 6:**
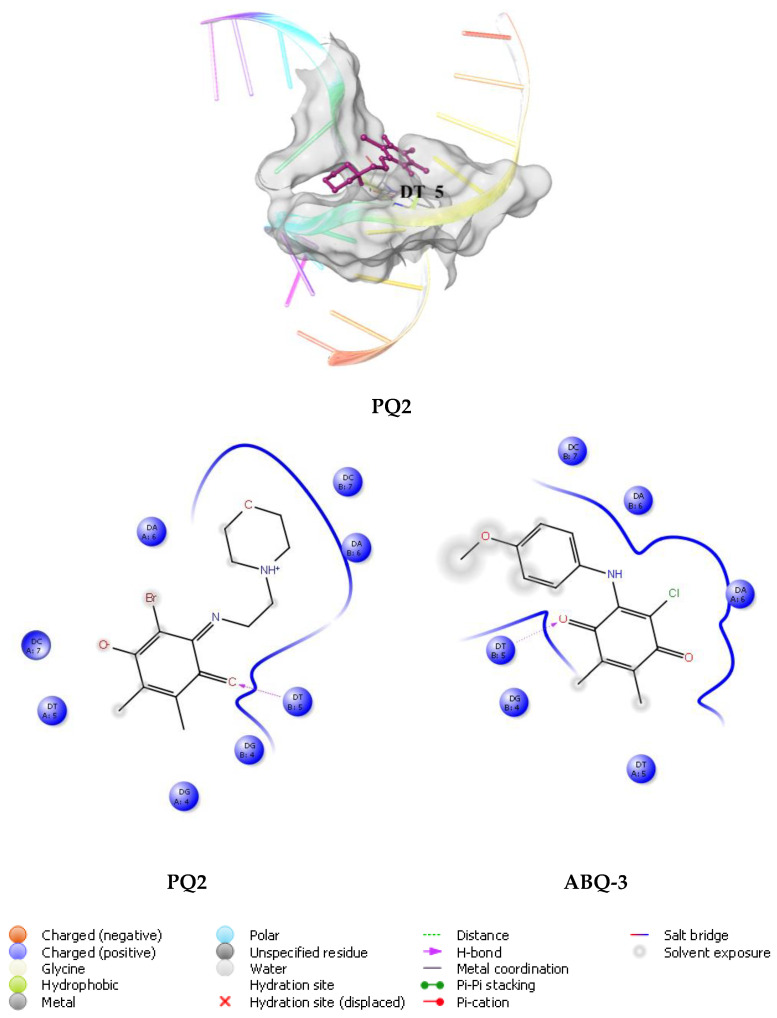
Docking pose and interactions of **PQ2** in the minor groove of the double helix of DNA (PDB ID: 2GWA) compared to docking interactions **ABQ-3** [64].

In our previous studies, we showed that quinone derivatives could bind DNA significantly [58,59,62,63,64]. Therefore, in the current work, molecular docking studies were carried out in the minor groove of the double helix of DNA (PDB ID: 2GWA) [61] to investigate the DNA binding effects of **PQ2**. Results manifested that **PQ2** revealed a high affinity for DNA *via* a key hydrogen bond with DT-5 (Figure 6). In our previous study [64], we also detected the same interaction with the aminobenzoquinone derivative (**ABQ-3**) (Figure 6). This finding emphasized the potential of the presence of aminobenzoquinone in the DNA-binding potential of these molecules.

During the drug development process, many drug candidates fail to pass the trials owing to their insufficient ADME (absorption, distribution, metabolism, and excretion) profiles. Computational ADME prediction is a rational and cost-saving analysis for searching for the drug-like properties of numerous compounds. In the current study, we conducted ADME analysis of **PQ2** using QikProp [65] and SwissADME [66] tools since it was identified as the most promising and selective anticancer agent. According to the results, **PQ2** revealed a remarkable pharmacokinetic profile with the key ADME parameters in acceptable ranges. **PQ2** exhibited appropriate lipophilicity and aqueous solubility with the octanol/water partition coefficient (QPlogPo/w) and predicted aqueous solubility (QPlogS) values, which were detected as 1.434 and −1.970, respectively (the limits: −2 to 6.5 for QPlogPo/w and −6.5 to 0.5 for QPlogS). The human serum albumin binding (QPlogKhsa) value, which is associated with the volume of distribution and half-life of drugs belonging to **PQ2**, was detected as −0.417 (the limit between −1.5 and 1.5). **PQ2** demonstrated significant gastrointestinal absorption (85%), which is referred to as >80% as high and <25% as poor. This compound violated the rules of Lipinski’s rule of 5 and Jorgensen’s rule of 3. The positive and acceptable brain/blood partition coefficient (QPlogBB) value of **PQ2** (0.135) estimated that this compound could cross the brain/blood barrier.

**Figure 7:**
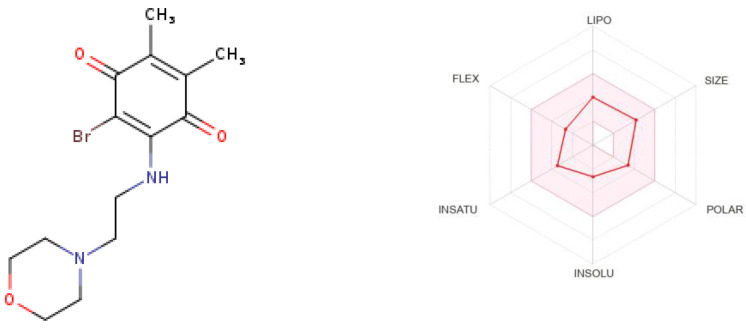
Bioavailability radar for **PQ2** from the SwissADME web tool.

**PQ2** provided all the limits for values of saturation (INSATU), size (SIZE), polarity (POLAR), solubility (INSOLU), lipophilicity (LIPO), and flexibility (FLEX) of the SwissADME bioavailability radar since its red distorted hexagon was involved within the pink area (Figure 7). In addition, **PQ2** matched the inhibition of several cytochrome P450 (CYP) enzymes, decreasing its potential to cause drug-drug interactions. All these pharmacokinetic parameters of **PQ2** indicated that **PQ2** showed drug-like properties for future studies.

## 4. Conclusions

In summary, starting from the anticancer product NSC 663284 from the NCI database, a number of nonhalogenated and halogenated PQ analogs as NSC 663284 analogs were designed and synthesized by scaffold hopping and further structural modifications within the quinone moiety. The successful scaffold hopping and further structural modifications of NSC 663284 led to the discovery of two novel potential anticancer lead molecules. Two of the obtained PQ analogs (**PQ2** and **PQ3**) were selected for the five-dose assay after preliminary screening by the National Cancer Institute’s Developmental Therapeutics Program. Both of them (**PQ2** and **PQ3**) exhibited excellent anticancer activity against some cancer cell lines, including leukemia, melanoma, ovarian cancer, and breast cancer subpanels. In particular, **PQ2** has the advantages of facile synthesis and strong inhibitory effects in leukemia cells. **PQ2** also exerted a cancer cell-selective action against the Jurkat cell line, displaying no toxicity on PBMC (healthy). Therefore, we performed further in silico studies for **PQ2**, focusing on Abl TK, one of the crucial targets of leukemia. **PQ2** was capable of forming key interactions similar to both dasatinib and imatinib, well-known Abl TK inhibitors. We also showed that **PQ2** could bind to the minor groove of the double helix of DNA, supporting our previous studies. Moreover, **PQ2** was endowed with drug-like properties based on ADME calculations. **PQ2** represents a good starting point for the development of a new generation of anticancer agents. For that reason, further studies on scaffold hopping and structural modification strategies are in progress in our laboratory.

## Figures and Tables

**Table 1:** Growth percentage results are per single-dose assay of the PQ analogs (**PQ1**–**4**).

Cancer Cell Lines		PQ1 (D-838754/1)	PQ2 (D-837715/1)	PQ3 (D-838755/1)	PQ4 (D-838758/1)
**Leukemia**					
GP	CCRF-CEM	98.14	−21.05	73.74	26.16
	HL-60(TB)	96.71	−24.93	10.26	68.81
	K-562	97.40	0.99	5.24	56.63
	MOLT-4	104.00	0.28	87.07	58.71
	RPMI-8226	92.90	58.59	88.36	85.40
	SR	96.65	55.91	99.69	99.98
**Non-Small Cell Lung Cancer**					
GP	A549/ATCC	110.25	87.57	102.61	104.88
	EKVX	95.10	46.44	58.55	75.59
	HOP-62	103.37	87.21	68.69	104.49
	HOP-92	98.81	38.69	114.18	107.70
	NCI-H226	97.55	67.08	27.88	94.68
	NCI-H23	95.02	−0.61	8.14	52.57
	NCI-H322M	101.36	91.51	103.91	98.10
	NCI-H460	103.89	97.97	102.17	103.40
	NCI-H522	92.26	−18.44	−39.17	96.16
**Colon Cancer**					
GP	COLO 205	111.45	129.82	−75.81	120.20
	HCC-2998	107.00	101.25	117.04	121.61
	HCT-116	107.87	−75.55	14.55	37.97
	HCT-15	108.15	90.72	97.45	107.40
	HT29	110.66	93.70	15.50	117.75
	KM12	99.66	94.18	99.89	100.91
	SW-620	95.25	32.71	−67.98	97.86
**CNS Cancer**					
GP	SF-268	96.23	63.68	111.69	105.60
	SF-295	105.00	103.06	91.72	99.71
	SF-539	95.10	32.06	106.82	104.23
	SNB-19	96.86	91.26	92.47	95.16
	SNB-75	nd	105.87	nd	nd
	U251	104.89	44.78	36.73	77.91
**Melanoma**					
GP	LOX IMVI	88.51	−28.50	−84.32	23.24
	MALME-3M	98.09	−68.96	−83.36	97.42
	M14	102.24	37.33	40.42	82.94
	MDA-MB-435	103.36	−87.50	−92.95	97.62
	SK-MEL-2	nd	68.89	nd	nd
	SK-MEL-28	109.52	75.37	51.44	119.00
	SK-MEL-5	94.12	62.08	84.40	75.65
	UACC-257	101.01	−13.26	−50.56	74.16
	UACC-62	94.97	39.37	−27.50	79.11
**Ovarian Cancer**					
GP	IGROV1	107.81	31.40	95.36	108.74
	OVCAR-3	100.23	−26.94	1.12	100.55
	OVCAR-4	100.84	−67.38	34.55	99.26
	OVCAR-5	111.14	4.00	5.49	113.98
	OVCAR-8	99.72	16.31	82.87	88.94
	NCI/ADR-RES	97.77	86.39	89.02	78.01
	SK-OV-3	94.20	87.54	83.08	92.47
**Renal Cancer**					
GP	786-0	107.58	100.01	104.36	106.45
	A498	117.40	123.55	139.17	138.52
	ACHN	98.19	62.27	83.07	93.00
	CAKI-1	92.79	45.31	85.40	94.84
	RXF 393	127.93	122.04	111.04	124.75
	SN12C	95.55	51.99	61.82	96.11
	TK-10	116.06	149.92	106.85	138.28
	UO-31	nd	44.75	nd	nd
**Prostate Cancer**					
GP	PC-3	93.40	61.79	82.26	83.31
	DU-145	107.42	−76.86	94.31	113.09
**Breast Cancer**					
GP	MCF7	97.84	10.33	−55.70	82.24
	MDA-MB-231/ATCC	nd	−6.27	nd	nd
	HS 578T	99.52	60.36	98.54	100.32
	BT-549	121.24	87.43	105.05	112.78
	T-47D	95.48	−58.60	44.01	−47.43
	MDA-MB-468	106.65	−96.19	−83.56	−89.03

nd means “not determined”.

**Table 2:** TGI and LC_50_ values (in µM) of anticancer activity data as per a five-dose assay of the selected PQ analogs (**PQ2** and **PQ3**) after 48 h based on the SRB assay at NCI.

Molecule	PQ2 (D-837715/1)			PQ3 (D-838755/1)		
Panel/Cell Line	GI_50_	TGI	LC_50_	GI_50_	TGI	LC_50_
**Leukemia**						
CCRF-CEM	3.44	>100	>100	19.8	53.6	>100
HL-60(TB)	2.46	7.24	>100	3.76	16.6	76.1
K-562	3.30	61.9	>100	4.25	60.8	>100
MOLT-4	5.35	32.8	>100	15.4	39.5	>100
RPMI-8226	13.9	55.0	>100	15.1	38.2	96.3
SR	19.2	66.7	>100	22.8	58.3	>100
**Non-Small Cell Lung Cancer**						
A549/ATCC	17.4	34.0	66.3	23.2	53.3	>100
EKVX	12.8	25.9	52.0	14.3	28.0	54.9
HOP-62	14.6	28.1	54.0	14.6	27.9	53.5
HOP-92	13.3	28.0	58.9	13.3	29.1	63.7
NCI-H226	12.1	26.7	59.2	15.6	33.6	72.4
NCI-H23	3.84	16.3	43.2	10.5	25.8	63.1
NCI-H322M	14.8	28.1	53.3	14.2	27.2	52.3
NCI-H460	16.1	34.2	72.6	17.1	36.1	76.2
NCI-H522	3.01	11.7	34.7	5.30	19.4	50.3
**Colon Cancer**						
COLO 205	17.9	33.6	63.4	17.6	31.9	57.8
HCC-2998	15.3	28.7	54.1	16.1	31.6	61.7
HCT-116	2.93	10.9	33.7	7.58	24.3	65.7
HCT-15	15.9	30.9	59.9	17.0	37.7	83.9
HT29	16.0	31.3	61.0	21.8	46.3	98.3
KM12	16.7	39.6	94.0	18.3	41.2	92.8
SW-620	2.94	14.0	52.3	13.9	29.4	62.1
**CNS Cancer**						
SF-268	12.8	32.0	80.2	17.1	31.9	59.8
SF-295	17.7	31.9	57.4	12.8	25.6	51.1
SF-539	17.0	31.5	58.2	11.9	24.5	50.4
SNB-19	16.3	30.5	57.2	16.0	29.7	54.9
SNB-75	18.5	34.2	63.0	11.0	23.0	48.2
U251	12.0	25.2	52.7	15.6	45.0	>100
**Melanoma**						
LOX IMVI	2.33	8.36	32.0	2.56	11.5	35.4
MALME-3M	2.25	5.37	17.2	1.71	3.31	6.41
M14	13.6	26.7	52.5	14.8	28.4	54.3
MDA-MB-435	1.81	3.35	6.21	1.72	3.18	5.88
SK-MEL-2	11.7	24.2	50.1	12.1	26.6	58.4
SK-MEL-28	14.5	28.0	54.0	8.19	20.9	46.7
SK-MEL-5	11.6	23.8	49.1	12.3	24.8	50.1
UACC-257	2.55	7.00	25.0	2.33	5.09	14.7
UACC-62	5.78	18.4	43.8	14.4	27.6	52.9
**Ovarian Cancer**						
IGROV1	10.5	22.9	49.9	12.7	26.3	54.2
OVCAR-3	2.10	7.16	29.1	2.06	6.51	24.8
OVCAR-4	5.02	17.6	42.7	8.18	20.9	46.3
OVCAR-5	6.43	19.3	44.8	3.65	14.7	43.7
OVCAR-8	3.84	15.8	48.0	18.4	40.3	88.4
NCI/ADR-RES	-	-	-	17.6	38.2	82.9
SK-OV-3	12.5	25.2	50.8	14.8	31.7	67.8
**Renal Cancer**						
786-0	17.8	32.1	58.1	18.3	32.8	58.8
A498	19.2	34.0	60.4	18.8	33.5	59.5
ACHN	13.8	26.8	51.8	12.5	25.1	50.2
CAKI-1	10.7	23.3	50.4	11.9	24.5	50.3
RXF 393	12.7	25.3	50.5	17.2	31.2	56.7
SN12C	14.9	28.3	53.5	-	-	-
TK-10	19.2	33.3	57.8	22.6	37.9	63.8
UO-31	11.0	23.2	49.1	8.31	21.7	48.0
**Prostate Cancer**						
PC-3	16.9	50.6	>100	16.5	66.4	>100
DU-145	12.7	25.7	52.0	2.31	5.67	18.6
**Breast Cancer**						
MCF7	3.66	15.2	50.9	1.80	3.91	8.53
MDA-MB-231/ATCC	5.65	18.7	47.1	13.9	32.0	73.80
HS 578T	9.04	34.6	>100	20.0	62.8	>100
BT-549	13.7	26.6	51.8	14.5	27.8	53.3
T-47D	2.01	4.74	69.7	12.3	33.4	91.3
MDA-MB-468	0.222	0.885	3.25	1.21	2.88	6.83

**Table 3:** The anticancer effects of **PQ2** on Jurkat cells and PBMC compared to imatinib.

Compound	Cell Type (IC_50_, μM)		SI *
	Jurkat	PBMC	
**PQ2**	2.94 ± 1.72	12.62 ± 4.07	4.29
Imatinib	7.11 ± 1.18	25.14 ± 6.41	3.54

* Selectivity Index (SI) = IC_50_ for PBMCs/IC_50_ for Jurkat cells.

## Data Availability

Data are contained within the article and Supplementary Materials.

## Supplementary Materials

The following supporting information can be downloaded at: https://www.mdpi.com/article/10.3390/biomedicines12010050/s1, Figure S1: ^1^H NMR (500 MHz) spectrum of the **PQ1** in CDCl_3_*-d1*; Figure S2: ^13^C NMR (125 MHz) spectrum of the **PQ1** in CDCl_3_*-d1*; Figure S3: ^1^H NMR (500 MHz) spectrum of the **PQ2** in CDCl_3_*-d1*; Figure S4: ^13^C NMR (125 MHz) spectrum of the **PQ2** in CDCl_3_*-d1*; Figure S5: ^1^H NMR (500 MHz) spectrum of the **PQ3** in CDCl_3_*-d1*; Figure S6: ^13^C NMR (125 MHz) spectrum of the **PQ3** in CDCl_3_*-d1*; Figure S7: ^1^H NMR (500 MHz) spectrum of the **PQ4** in CDCl_3_*-d1*; Figure S8: ^13^C NMR (125 MHz) spectrum of the **PQ4** in CDCl_3_*-d1*; Figure S9: HRMS spectrum of the **PQ1**; Figure S10: HRMS spectrum of the **PQ2**; Figure S11: HRMS spectrum of the **PQ3**; Figure S12: HRMS spectrum of the **PQ4**.
