# Supplementary material for: Scaffold Hopping and Structural Modification of NSC 663284: Discovery of Potent (Non)Halogenated Aminobenzoquinones
Source: Biomedicines. 2023 Dec 24;12(1):50. doi: 10.3390/biomedicines12010050 (PMC10813041; doi:10.3390/biomedicines12010050)
Supplement: Supplementary file 1 [file biomedicines-12-00050-s001.zip › biomedicines-2753279-supplementary.pdf]

Supplementary Material (SM)

Scaffold Hopping and Structural Modification of NSC 663284: Discovery of Potent (Non)Halogenated Aminobenzoquinones

Nilüfer Bayrak [1], Belgin Sever [2,3], Halilibrahim Ciftci [3,4,5], Masami Otsuka [3,4], Mikako Fujita [3] and Amaç Fatih TuYuN [1,*]

[1] Department of Chemistry, Faculty of Science, Istanbul University, Fatih, İstanbul 34126, Turkey; nbayrak@istanbul.edu.tr
[2] Department of Pharmaceutical Chemistry, Faculty of Pharmacy, Anadolu University, Eskisehir 26470, Turkey; belginsever@anadolu.edu.tr
[3] Medicinal and Biological Chemistry Science Farm Joint Research Laboratory, Faculty of Life Sciences, Kumamoto University, Kumamoto 862-0973, Japan; hiciftci@kumamoto-u.ac.jp (H.C.); motsuka@gpo.kumamoto-u.ac.jp (M.O.); mfujita@kumamoto-u.ac.jp (M.F.)
[4] Department of Drug Discovery, Science Farm Ltd., Kumamoto 862-0976, Japan
[5] Department of Molecular Biology and Genetics, Koc University, Istanbul 34450, Turkey
* Correspondence: aftuyun@gmail.com or aftuyun@istanbul.edu.tr; Tel.: +90-212-440-0000

Contents

^{1}H and ^{13}C spectra of the PQ analogs (**PQ1-4**) S3-6

HRMS spectra of the PQ analogs (**PQ1-4**) S7

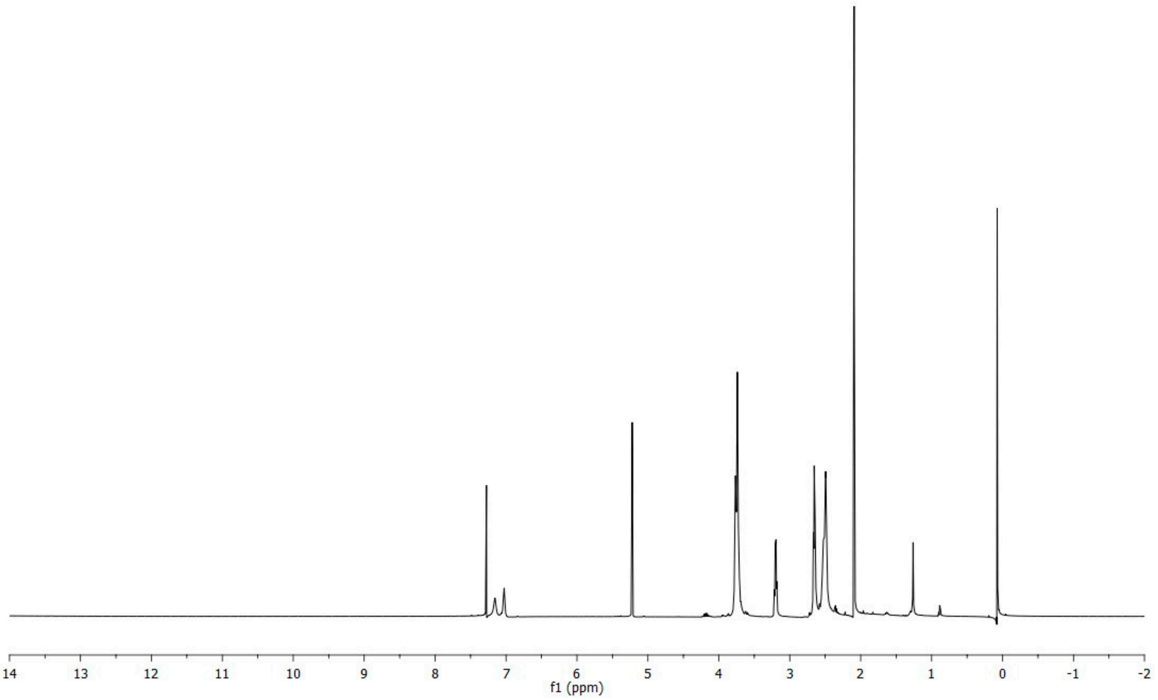

Figure S1: ^{1}H NMR (500 MHz) spectrum of the **PQ1** in $CDCl_3$-*d1*

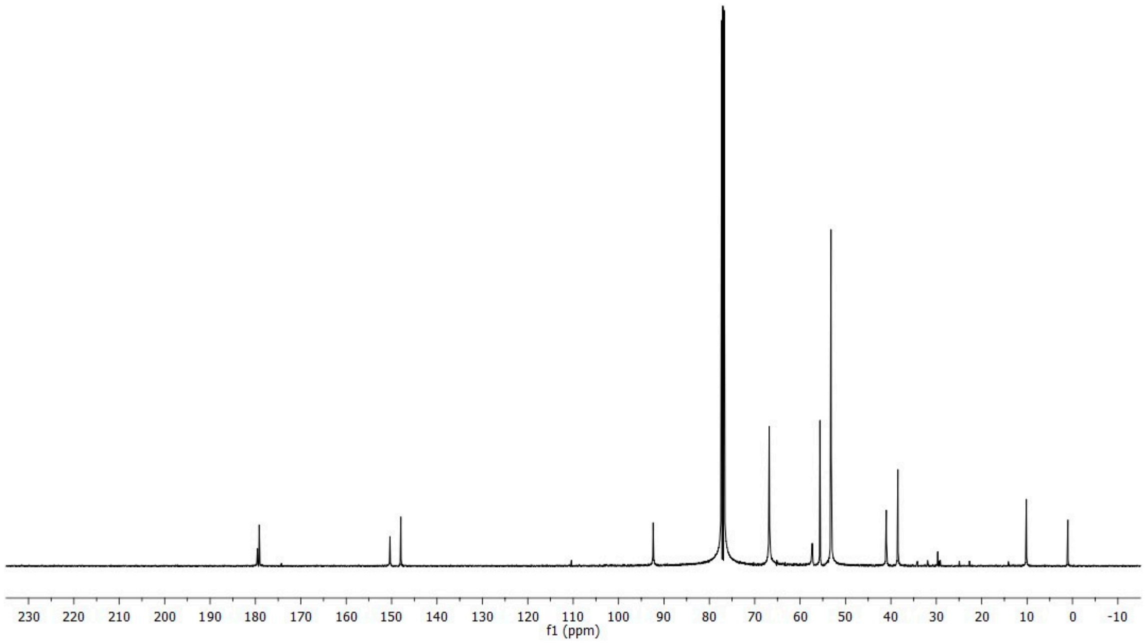

Figure S2: ^{13}C NMR (125 MHz) spectrum of the **PQ1** in $CDCl_3$-*d1*

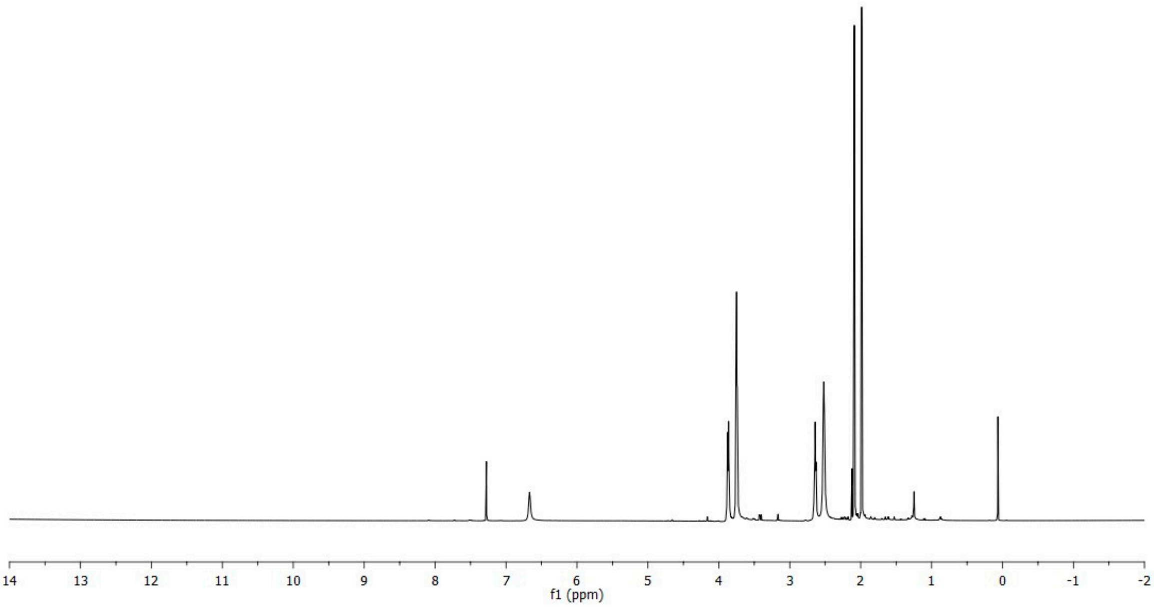

Figure S3: ^{1}H NMR (500 MHz) spectrum of the **PQ2** in $CDCl_3$-*d1*

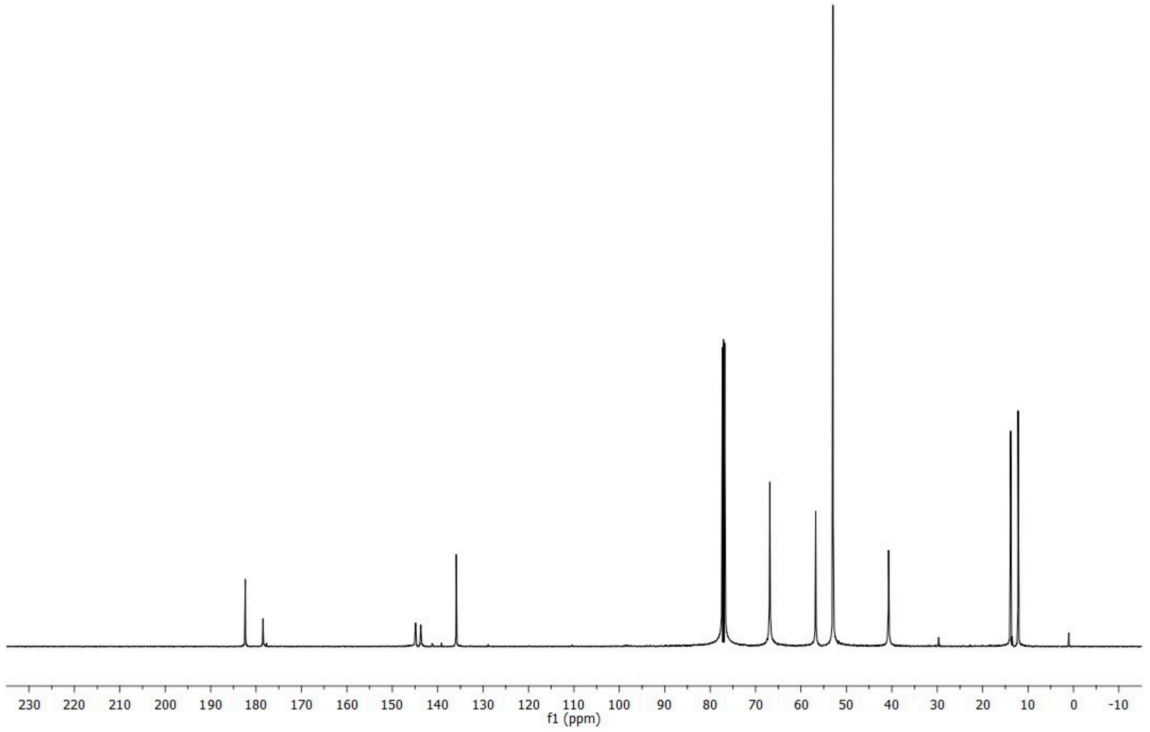

Figure S4: ^{13}C NMR (125 MHz) spectrum of the **PQ2** in $CDCl_3$-*d1*

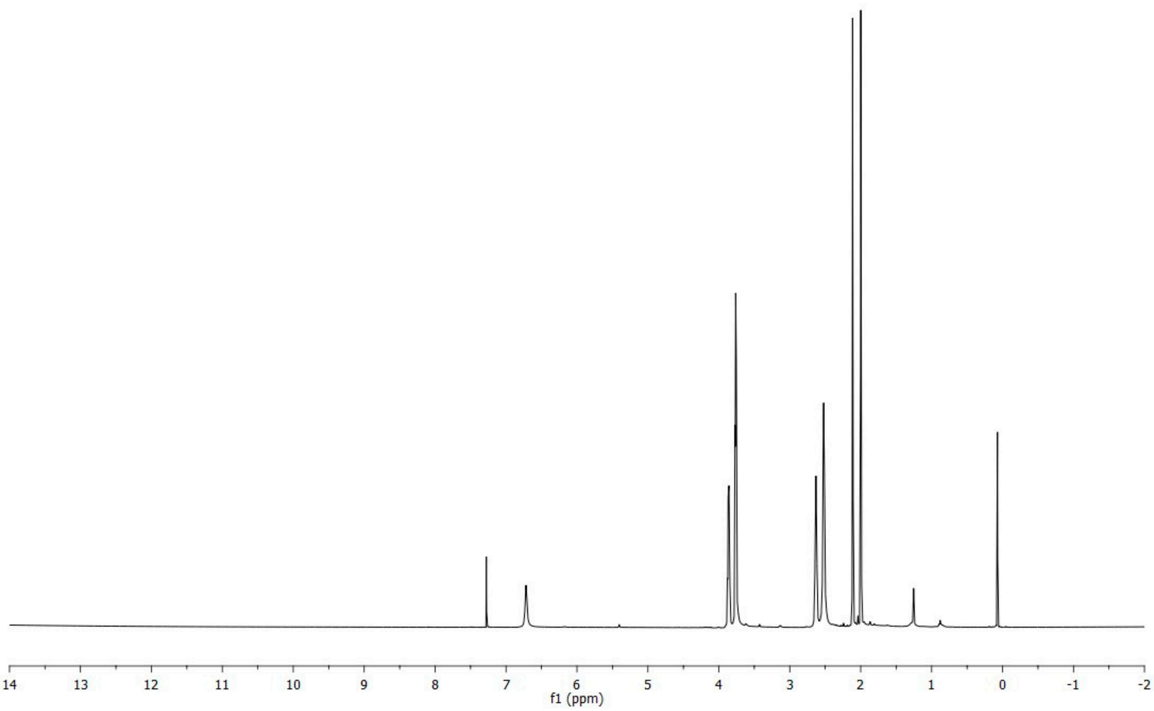

Figure S5: ^{1}H NMR (500 MHz) spectrum of the **PQ3** in $CDCl_3$-*d1*

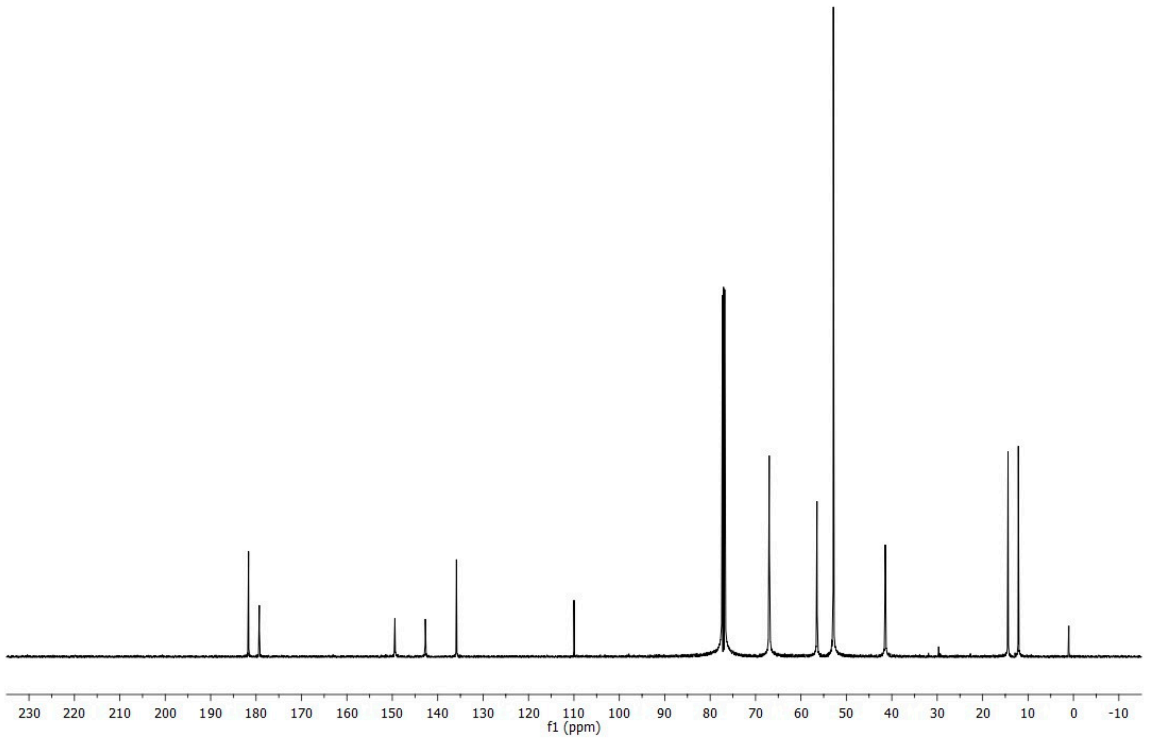

Figure S6: ^{13}C NMR (125 MHz) spectrum of the **PQ3** in $CDCl_3$-*d1*

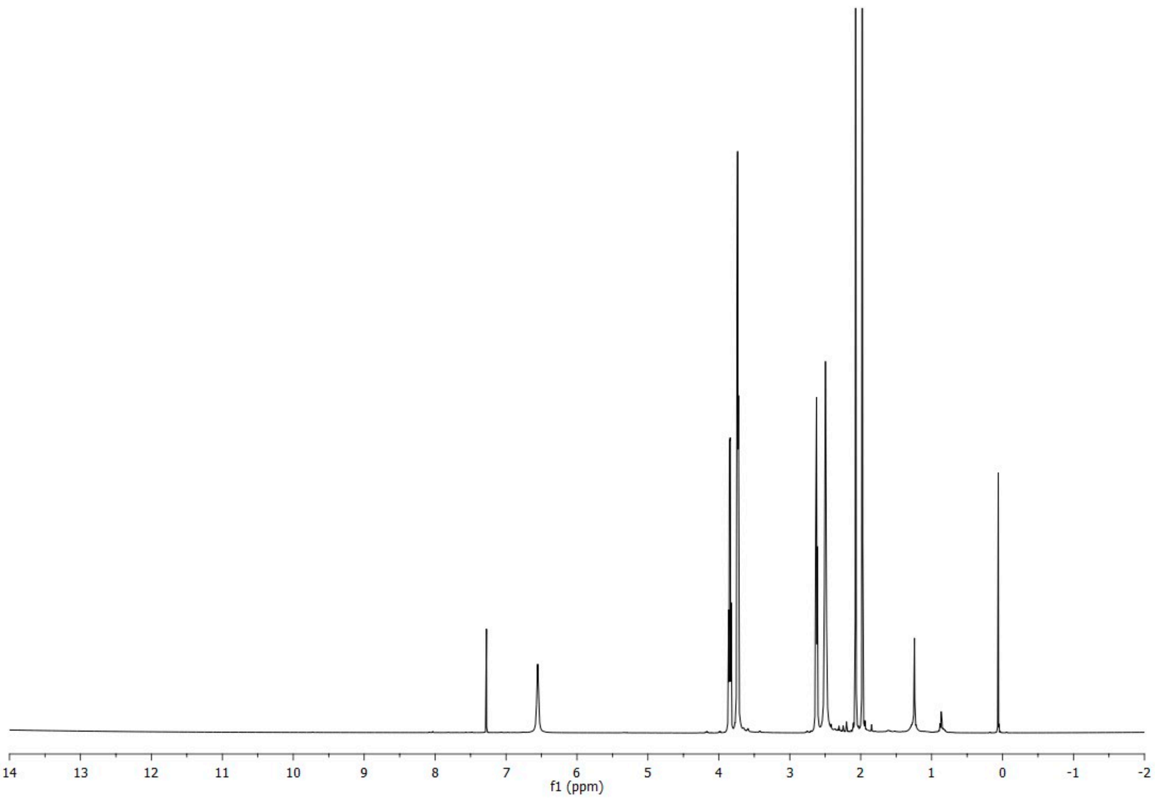

Figure S7: ^{1}H NMR (500 MHz) spectrum of the **PQ4** in $CDCl_3$-*d1*

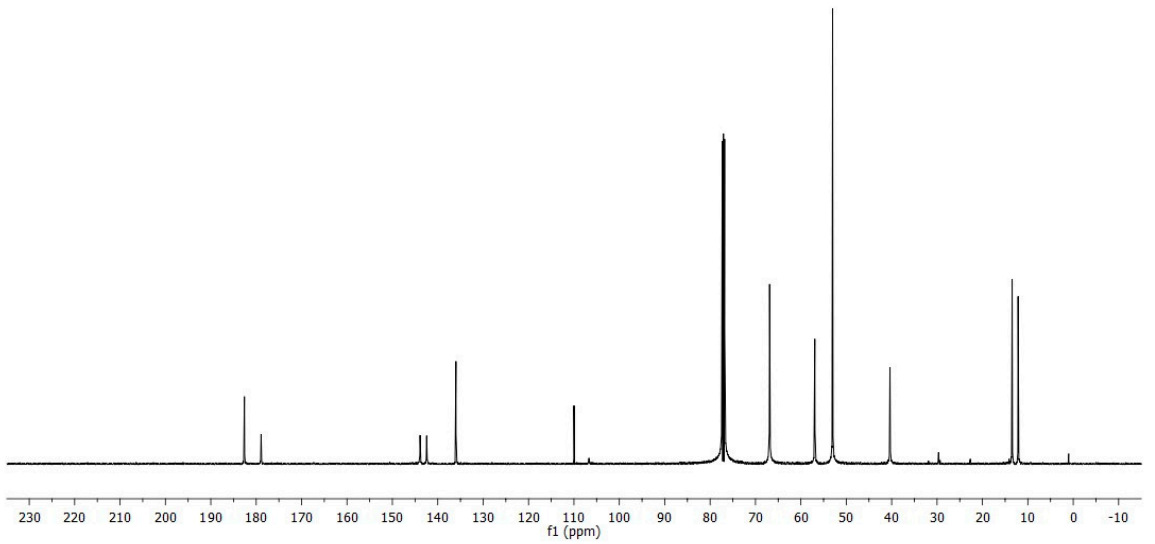

Figure S8: ^{13}C NMR (125 MHz) spectrum of the **PQ4** in $CDCl_3$-*d1*

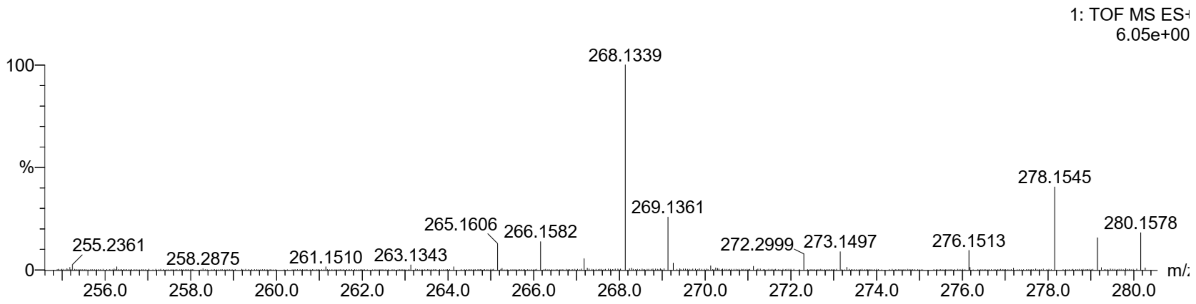

Figure S9: HRMS spectrum of the **PQ1**

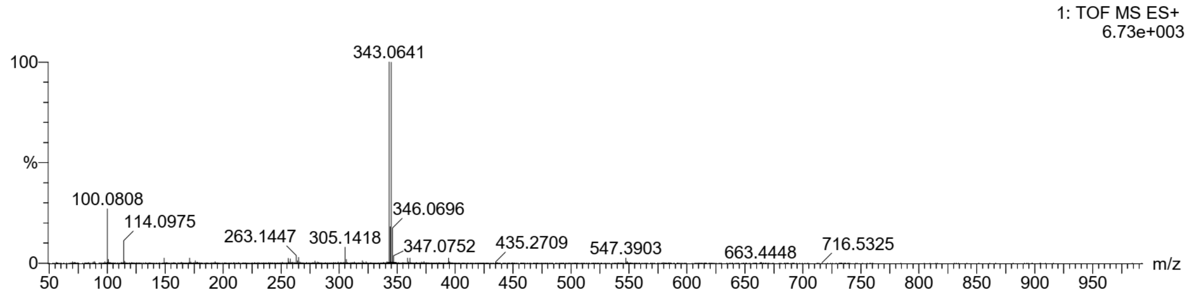

Figure S10: HRMS spectrum of the **PQ2**

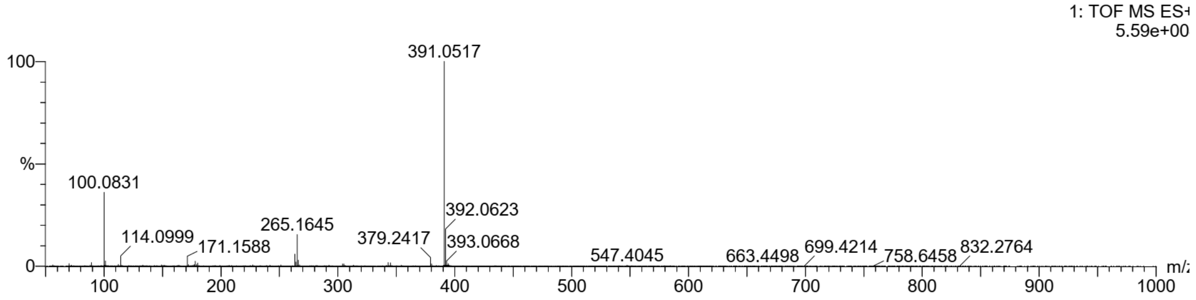

Figure S11: HRMS spectrum of the **PQ3**

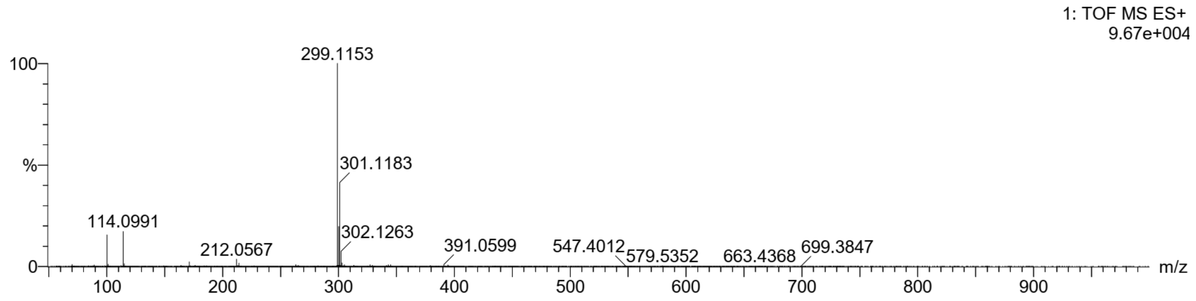

Figure S12: HRMS spectrum of the **PQ4**
